# Use of Lisdexamfetamine to Treat Obesity in an Adolescent with Severe Obesity and Binge Eating

**DOI:** 10.3390/children6020022

**Published:** 2019-02-04

**Authors:** Gitanjali Srivastava, Valerie O’Hara, Nancy Browne

**Affiliations:** 1 Section of Endocrinology, Diabetes, Nutrition & Weight Management, Department of Medicine, Boston University School of Medicine, 720 Harrison Avenue, Suite 801, Boston, MA 02114, USA; 2 Boston Medical Center, Nutrition and Weight Management Center, 720 Harrison Avenue, Suite 801, Boston, MA 02114, USA; 3 Eastern Maine Medical Center, Department of Pediatrics, WOW Pediatric & Adolescent Weight & Cardiometabolic Clinic, Orono, ME 04401, USA; vohara@northernlight.org (V.O.); nbrowne@northernlight.org (N.B.)

**Keywords:** severe pediatric obesity, adolescent obesity, pharmacotherapy, lisdexamfetamine, BMI percentile, weight loss medication, pediatric weight management

## Abstract

Approximately two-thirds of US children and adolescents have either obesity or overweight status, with almost 24% of adolescents (ages 12–19 years) afflicted with severe obesity, defined as >1.2 × the 95th BMI percentile for age/gender. Despite the increasing disproportionate rise in severe or extreme childhood obesity, many children in weight management programs do not achieve a healthy weight. Most often, these patients will go on to require metabolic and bariatric surgery (MBS), but challenges and limitations may prohibit MBS on adolescents. Thus, tertiary care pediatric weight management centers are compelled to treat select pediatric obesity subtypes presenting with disease progression and disability with the available adult FDA-approved therapeutic modalities, specifically pharmacotherapy, in order to alleviate the disease state and provide relief to the patient. Here, we describe a case of severe pediatric obesity where a dedicated multidisciplinary pediatric weight management team at a tertiary care center utilizes a progressive pharmacotherapeutic approach with enormous benefits to the patient, highlighting the urgent gap and clinical care needs of this special population niche of severe adolescent obesity.

## 1. Introduction

Severe obesity in adolescents has risen disproportionately in the United States over the past 30 years [1]. As teenagers, most of these patients are already affected by detrimental health effects as a result of the disease process, wherein inflammatory pathophysiology mimics more closely that of an affected adult with cardiovascular disease and diabetes [2,3]. The treatment options are limited: [1] intensive lifestyle modification therapy (ILT) or [2] metabolic and bariatric surgery (MBS), an option for only a few subsets of adolescents due to challenges and limitations in access and coverage [4,5]. ILT outcomes are disappointing as a majority of adolescents with severe obesity, despite repeated efforts, are non-responders or achieve only a modest effect on weight with some underlying improvement of metabolic parameters [6,7]. Although anti-obesity pharmacotherapy is approved for the chronic treatment of adults with obesity and often serves as a bridge between ILT and MBS in the adult population afflicted with obesity [8], there are very few studies of anti-obesity medications in pediatrics and adolescent patients [4]. In this case report, we describe the use of lisdexamfetamine treatment on an adolescent patient with severe obesity, binge eating disorder and attention deficit disorder. Ultimately, the decision to consider an off-label non-FDA-approved medication (lisdexamfetatmine)—also a controlled substance—for the treatment of severe pediatric obesity, in the setting of a binge eating disorder with an associated comorbid diagnosis, rested on the prudent multidisciplinary team—well trained in clinical anti-obesity therapeutic applications. Overarching benefits were achieved after years of attempts to treat the chronic disease of obesity.

## 2. Case Presentation

A 9-year-old girl was referred to a tertiary care pediatric weight management clinic (PWMC) by her primary pediatrician for worsening obesity, with a BMI of 32.5 kg/m^2^ (1.4 × the 95th BMI percentile for age/gender). The patient’s weight progressed to the higher end of the pediatric growth curve early in life. For 2 years prior to presentation at PWMC, the patient’s weight had been of highest concern with perceived triggers related to parental divorce, the stress of two households, and emotional eating. Her lipid levels were significantly elevated. The patient had a moderate activity level (school physical education classes, horse-riding therapy, and daily walking). Her diet included frequently eating outside the home, drinking soda every other day, and low intake of fruits and vegetables. Water intake was limited. The patient’s parents (divorced) shared custody and the patient spent time in two households during the week. Her screen time averaged 3 h per day. 

Birth history revealed a normal vaginal delivery without complications with a birth weight of 3.95 kg (large for gestational age) and length of 50.80 cm. The patient underwent neuropsychiatric evaluation at the age of 6 years for developmental delay and autism spectrum diagnoses; physical and occupational therapies were provided through early school age years. Her family history was positive for obesity on the maternal side; diabetes, heart disease and hyperlipidemia were diagnosed on both paternal and maternal lineages.

A review of the patient’s systems revealed normal menarche and menstrual cycles. The patient denied snoring, headaches and frequent nocturnal awakenings, although she admitted to restless sleep and feeling tired.

Her vital signs were normal for her age, gender and height (BP 110/50, pulse 88) with a normal physical examination except for central obesity in the absence of lipodystrophy. There was no evidence of the syndromic features of obesity or acanthosis nigricans. Mild developmental delay was present as documented by prior evaluations. Between the ages of 9 and 16 years, the patient was continuously followed in PWMC, with focus around intensive lifestyle treatment. By the age of 16 years, the patient’s weight had approached 273.8 pounds with a height of 63 inches and a BMI of 48.68 (1.58 × the 95th BMI percentile; Class 3 obesity), BP of 116/70 (normal range for age/gender/height), and Tanner stage 5.

Laboratory evaluation at initial presentation revealed total cholesterol of 375 mg/dL, LDL 293 mg/dL, triglycerides 125 mg/dL, HDL 57 mg/dL, fasting blood glucose of 90 mg/dL, TSH 2.83 mIU/L, fasting insulin level of 10 uU/mL, vitamin D-25-hydroxy level of 17 ng/mL, and ALT 15/AST 18 units/L. A repeat laboratory panel after the patient was started on a statin medication (simvastatin 10 mg increased over time to 40 mg) by her lipid specialist showed an overall improvement in her lipid profile (in mg/dL): total cholesterol of 203, LDL 137, triglycerides of 62, and HDL 54. Vitamin D-35-hydroxy levels had improved to 28 ng/mL with supplementation. Repeat fasting blood glucose and hemoglobin A1c remained stable as a teenager at 88 mg/dL and 5.4% respectively. A polysomnogram at the age of 15 years showed an apnea-hypopnea-index (AHI) of 1.1, indicative of mild sleep apnea.

### 2.1. Assessment and Plan

A 16-year-old girl with severe childhood obesity (>1.4 × the 95th BMI percentile) since the age of 9 years, with related comorbidities and resistance to ILT, presented to the PWMC. Since initial presentation to the PWMC, the patient progressively deteriorated during adolescence despite ILT in a tertiary care pediatric weight management center. The significant psychosocial barriers to care included the diagnosis of developmental delay/autism and milieu instability.

**Figure 1:**
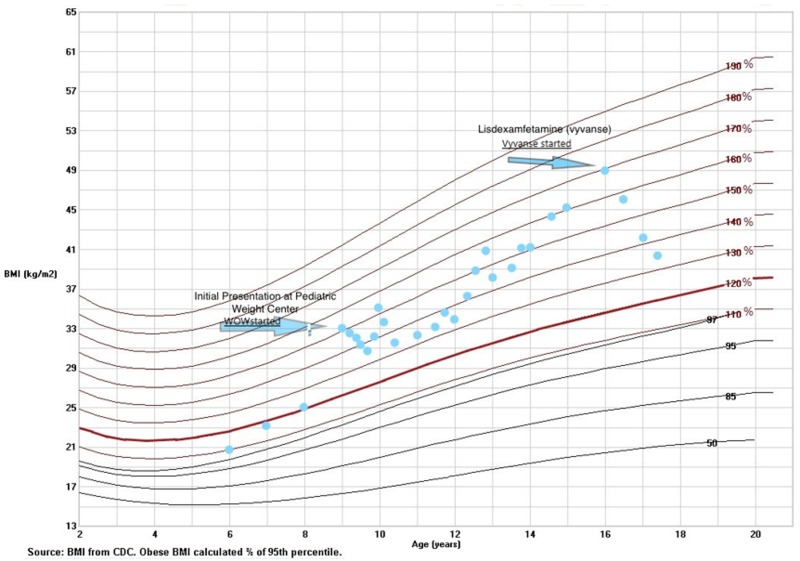
Body Mass Index (BMI) percentile growth curve for the patient in the case report at presentation (first arrow) and with treatment (second arrow).

The initial treatment at PWMC involved ILT goals focusing on replacing sugar sweetened beverages with water and zero sugar options, building on physical activity, increasing fruit/vegetable consumption as well as MyPlate model [9] for portion sizes. Screen time and the consistency of time between households were addressed with emphasis on limiting processed food choices when eating outside the home. The BMI% trajectory trend showed stabilization over the first year. However, despite ILT, the patient’s BMI% continued to demonstrate an upward incline after the age of 10.5 years (Figure 1). 

The overall minimal response to ILT and upward trends on BMI% were again discussed with the family when the patient reached 16 years of age. Prior records were reviewed including the early neuropsychiatric evaluation and current school performance. Despite some resource assistance at school, the parents and patient both noted increased anxiety at school as demands increased with school year progression. The signs and symptoms of emotional eating were then addressed. The patient’s mother reported that when the patient was old enough to walk home from school, she often stopped at the local store to buy food. The patient’s mother also noted that she limited food shopping as multiple days of food were being consumed within a day or two. The evaluation of binge eating disorder (BED) using the BED scale [10] and Attention Deficit Disorder (ADD) using the Vanderbilt scale [11] revealed positive results of both tests and confirmed the diagnosis respectively (the BED score of 21 and the Vanderbilt score indicated Attention Deficit-Inattentive type). The patient was evaluated and followed closely with a pediatric psychologist/developmental specialist and underwent concurrent cognitive behavioral therapy.

### 2.2. Re-Evaluation and New Plan: Decision to Inititate Lisdexamfetamine Therapy

Given the disease progression coupled with the comorbid obesity-related conditions and the limited response to ILT, the PWMC team felt that a more aggressive treatment plan of the patient’s severe obesity was indicated and imperative. Although the patient met the criteria for MBS [5], the patient and her mother declined surgical intervention despite engaging in discussions on this topic. Moreover, MBS services were non-existent within a 200 mile radius and surgical intervention would require significant travel to the nearest adolescent surgery tertiary care center. As a result, PWMC clinicians opted for a pharmacotherapy trial of lisdexamfetamine to address both BED and ADD in the setting of severe adolescent obesity, initially starting at the lowest dose of 20 mg once daily as recommended for ADD. The only two FDA-approved anti-obesity medications with concurrent pediatric indications (orlistat and phentermine) and other available off-label and FDA-approved anti-obesity pharmacotherapy for adults [8] (metformin, topiramate, zonisamide, naltrexone SR/buproprion SR, lorcaserin, liraglutide 3.0 mg) did not meet the requirements needed to address both BED and ADD. Significant family counseling discussing the pros and cons of using lisdexamfetamine, including medical literature references, occurred during multiple visits at PWMC, and all the risks were discussed and side effects were reviewed. Off-label consent was documented for BED, although the patient met criteria for use of this medication due to her ADD diagnosis [4,12]. Table 1 tracks the progression of her weight, dosage changes, and clinical changes over time on lisdexamfetamine. After one year of treatment on lisdexamfetamine, the patient lost a total of 39.4 lbs, with a reduction of BMI from 48.49 to 40.91 (decrease from 1.65 × to 1.35 × the 95th BMI percentile) on 50 mg lisdexamfetamine once daily (Figure 1 and Table 1). Repeat polysomnograms showed improvements in AHI down to 0.3 (previous 1.1) and repeat BED scales showed improvement from 21 to 5. The patient reported significant improvements in school performance and social anxiety. Pharmacotherapy, as an adjunct to healthier eating habits and lifestyle, helped reduce binge eating episodes as reported by the patient and family.

### 2.3. Consent for Publication

Written informed consent was obtained from the patient for the publication of this article and accompanying table.

## 3. Discussion

Approximately two-thirds of US children and adolescents experience overweight or obesity, with almost 24% of adolescents (ages 12–19 years) afflicted with severe obesity (Class 2 (1.2 ×) and Class 3 (1.4 × the 95th BMI percentile)) [1]. Disease severity correlates with worsening obesity-related comorbidities such as diabetes, cardiovascular disease, hypertension, and sleep apnea, overall quality-of-life and disability [3,13,14,15]. Moreover, the association between obesity and Attention Deficit Hyperactivity Disorder (ADHD) and BED is well-known [16,17]. Intensive lifestyle modification is often disappointing once the obesity disease state is established and escalation to more aggressive therapeutic intervention becomes urgent [18,19]. Although a majority of these patients may qualify for adolescent MBS [5], significant challenges such as psychosocial barriers, the availability of resources within rural areas and limited insurance reimbursement might be prohibitive. Thus, anti-obesity pharmacotherapy may serve as a bridge between intensive lifestyle therapy and metabolic and bariatric surgery in cases of severe childhood obesity [4,6]. Furthermore, anti-obesity pharmacotherapy can provide improvements in health-related quality of life in patients with obesity and overweight status long-term [20]. 

Unfortunately, there are currently only two available FDA-approved anti-obesity medications with pediatric indications—(1) orlistat approved for age ≥12 years and (2) phentermine for age >16 years—despite the availability of four other major FDA-approved anti-obesity medications (liraglutide 3.0 mg, naltrexone SR/bupropion SR, lorcaserin, phentermine/topiramate extended-release combination) [8]. The safety and long-term efficacy of these agents are questionable due to limited pediatric data and sparse pediatric clinical trials related to anti-obesity pharmaceuticals. Neither of these medications are approved for binge eating disorder in children, although lisdexamfetamine recently received FDA approval for the treatment of binge eating disorder in adults. Interestingly, lisdexamfetamine is approved for the long-term treatment of ADHD in children and adolescents ages 6–17 years [21]. In this case report, note that lisdexamfetamine was actually indicated for ADHD, provided double benefits, and off-label consent might not have been required. However, the pediatric team decided to obtain off-label medication use consent for BED regardless, highlighting the need for more research to guide clinical care and standardization of pediatric obesity care pathways. More recently, new recommendations for using obesity pharmacotherapy in children and adolescents have been proposed [4].

Lisdexamfetamine is a central nervous system amphetamine compound with high potential for abuse and dependence [21]. It is FDA-approved for ADHD in children/adolescents and BED in adults. Serious cardiovascular reactions such as sudden death have been reported in pediatric patients with structural cardiac abnormalities or other serious heart problems. In adults, sudden death, stroke or myocardial infarction have also been reported. Thus, the medication is absolutely contraindicated in cases of cardiovascular condition or disease states. Blood pressure and heart rate monitoring is advised along with monitoring for occurrence of depression exacerbation or mood changes. Suppression of growth without rebound may occur in pediatric patients (average height decline of ~2 cm less over 12 months and 2.7 kg less growth in weight over 2 years). The most common adverse reactions in children, adolescents, and/or adults with ADHD were anorexia, anxiety, decreased appetite, decreased weight, diarrhea, dizziness, dry mouth, irritability, insomnia, nausea, upper abdominal pain, and vomiting. These were similar in adults with binge eating disorder prescribed lisdexamfetamine [21].

Compared to the other obesity pharmacotherapeutic options utilized in adults, lisdexamfetamine has a long, safe history of use in children [22] and thus the PWMC team was more comfortable in its clinical application. However, despite the use of lisdexamfetamine in our patient and the absolute benefit it provided for the treatment of both binge eating disorder and severe obesity, we must caution readers against long-term use of lisdexamfetamine to treat obesity. The prescriptive therapy was delivered by trained providers in a multidisciplinary controlled setting well-versed in informed consent regulations, safety profiles, contraindications, and obesity subtypes. The patient was established with the clinical team for numerous years with documented efforts of intensive lifestyle modification therapy. Furthermore, practitioner prescribing of an off-label controlled substance in a pediatric patient might raise associated medical–legal issues in some States. Our patient also had a diagnosis of ADD which falls within legal regulations for using lisdexamfetamine. At this time, due to limited long-term data in children and adolescents, pediatric obesity pharmacotherapy clinical trials are much needed.

## 4. Conclusions

The case report underscores the importance of more evidence-based therapeutic options for severe obesity in adolescents. Despite disease prevalence and severity, treatment options for severe pediatric obesity remain scant and limited. Robust pediatric clinical trials endorsing the potential benefits of treatment, given the adverse effects of the disease, are necessary. Standardized road maps guiding clinical care on pharmacotherapeutic monitoring, assent and consent from parents for a chronic condition with long-term complications, along with appropriate screening tools, are needed to perpetuate forward momentum for the treatment of severe pediatric obesity. Finally, efforts to address this urgent need for the treatment of severe childhood obesity are underway [4]*,* but much work remains to be done, including clinical trials to evaluate the effectiveness of single- (ILT, pharmacology, medical devices, surgery) and multi-modal therapy in patients with select disease phenotypes of obesity.

## Figures and Tables

**Table 1:** Summary of the patient’s consecutive visits at the pediatric weight management center over time.

	Weight (lbs)	BMI	Lisdexamfetamine Dose	Side Effects	Clinical Progress and Comments
First visit Time 0	275	48.89 (165th BMI%)	20 mg once daily		Started at lowest 20 mg dose as recommended for ADD to monitor response and side effects. Family was more comfortable starting at the lowest dose. Dose would need to be titrated up in order to provide effect.
2 weeks	264	46.93	20 mg once daily	None	Patient was "beaming" and shared many positive correlations: easier choosing healthier options, decrease in snacking behavior when home alone, and improved focus on school and tasks.
1 month	257.4	45.76	Dose adjusted to 30 mg once daily	None	Patient was consuming less soda. She was applying for a job for the first time. She noted smaller portion sizes and a decrease in snacking behavior. However, dose was becoming less effective by afternoon or early evening.
2 month	253.6	45.09	30 mg once daily	None	Using FitBit to track physical activity and now rarely drinking soda.
3 months	253.8	45.10	30 mg once daily	None	Feeling more confident at school and with implementing ILT goals. Transitioned to a new school and tried out for a school play.
4 months	253.4	45.06	30 mg once daily	None	BMI had approached stability. The patient was off the medication for 1 week with return of her symptoms and increase in soda intake. Compliance and environmental triggers were reviewed.
4.5 months	244.6	43.49	30 mg once daily	None	Happier at school and making new friends.
6 months	242.8	43.17	30 mg once daily	None	Nominated Student of the Month. School grades had improved. Cravings were now well controlled. Repeat polysomnograms showed improvement in AHI down to 0.3 (previous 1.1). Repeat BED scales showed improvement from 21 to 5.
7.5 months	240	41.67	30 mg once daily	None	Patient continued to show stability.
8.5 months	241.6	41.95	30 mg once daily	None	Mild compliance issues with medication (missing one dose/week on average) with notable differences and worsening cravings upon discontinuation of the medication.
10 months	240	41.67	30 mg once daily	None	Stable progress noted.
11 months	242.8	42.16	Dose increase to 40mg once daily		Patient is now 17 years and 2 months old. Increase in soda consumption with bullying recently addressed directly with school official independently. Planning ahead with expected changes to summer schedule.
12 months	238.4	41.39	40 mg once daily	None	Increased compliance noted with improved physical activity and healthier choices. Applying for driver’s permit.
13 months	235.6	40.90	40 mg once daily	None	"I think the medication helps me tremendously." Decrease in binge eating patterns, cravings, hunger and more focus with school/home tasks. No longer at alternative locations, therefore less triggers.
15 months	239.6	41.60	40 mg once daily	None	Temporal anxiety and stressors noted. Referral to pediatric weight management psychologist with improvements noted.
16 months	233.6	40.84	40 mg once daily	None	Temporal stressors and anxiety alleviated.
17 months	235.2	40.84	Dose increase to 50mg once daily	None	Stressors and anxiety related to senior year high school schedule and increase in emotional eating.
18 months	235.6	40.91 (135th BMI%)	50 mg once daily	None	Graduating from high school and accepted into local college.
